# Alcohol-Related Content of Animated Cartoons: A Historical Perspective

**DOI:** 10.3389/fpubh.2013.00002

**Published:** 2013-03-28

**Authors:** Hugh Klein, Kenneth S. Shiffman

**Affiliations:** ^1^Kensington Research InstituteSilver Spring, MD, USA; ^2^Cable News NetworkAtlanta, GA, USA

**Keywords:** animated cartoons, media content, alcoholic beverages, drinking, portrayals, messages

## Abstract

This study, based on a stratified (by decade of production) random sample of 1,221 animated cartoons and 4,201 characters appearing in those cartoons, seeks to determine the prevalence of alcohol-related content; how, if at all, the prevalence changed between 1930 and 1996 (the years spanned by this research); and the types of messages that animated cartoons convey about beverage alcohol and drinking in terms of the characteristics that are associated with alcohol use, the contexts in which alcohol is used in cartoons, and the reasons why cartoon characters purportedly consume alcohol. Approximately 1 cartoon in 11 was found to contain alcohol-related content, indicating that the average child or adolescent viewer is exposed to approximately 24 alcohol-related messages each week just from the cartoons that he/she watches. Data indicated that the prevalence of alcohol-related content declined significantly over the years. Quite often, alcohol consumption was shown to result in no effects whatsoever for the drinker, and alcohol use often occurred when characters were alone. Overall, mixed, ambivalent messages were provided about drinking and the types of characters that did/not consume alcoholic beverages.

## Introduction

Data collected in recent years indicate that alcohol use is fairly widespread in the youth population and that drinking beyond initial experimentation is commonplace. More than one-third (35.8%) of the 13–14-year olds reported having used alcohol at least once in their lives (1). The comparable figures for 15–16 and 17–18-year olds are 58.2 and 71.0%, respectively. Alcohol-related intoxication also appears to be fairly widespread among adolescents and to become a more frequent occurrence as people progress through adolescence. Johnston et al.’s (1) data indicate that nearly one-half (45.5%) of the 13–14-year olds who reported having used alcohol had drunk to the point of intoxication. This compares to more than one-half of the 15–16-year olds studied (63.4%) and more than three-quarters of the 17–18-year olds studied (76.2%).

Given that the initiation of alcohol use usually occurs at an early age, and given that beliefs, attitudes, and knowledge about behaviors are generally considered to precede the initiation of the actual behaviors themselves (2), the preceding figures suggest that most of our nation’s children and adolescents form specific beliefs and attitudes about drinking while they are in childhood and/or adolescence. This assumption is based on the notion that it is their beliefs and attitudes about the use of alcohol that lead them to experiment with alcohol. Indeed, some researchers have reported that the precursors of actual drinking behaviors are well under development by the time a child reaches the third grade (3, 4), perhaps even as early as the first grade (5). This leaves two questions unanswered: What are the sources of young people’s information (or misinformation), beliefs, and attitudes regarding the use of alcohol? What induces them to experiment with drinking?

Numerous influences can be identified, including, among others, peer behaviors and peer pressure; curiosity; role modeling by parents and other authority figures; and the media. Regarding the latter, a large majority (88%) of the media effects studies conducted during the past few decades have learned that exposure to media messages, particularly visual electronic media messages, leads to changes in people’s beliefs, attitudes, and/or behaviors (6). Typically, these studies have contended that the effects produced in viewers of televised material are likely to be secondary to those provided by other, more influential sources in the viewers’ lives, such as family members, peers, and their own past experiences (7, 8, 9, 10). It is important to note, however, that not all researchers have found mass media effects to be of lesser strength than those provided by other sources in people’s lives. Atkin et al. (11) and Hollender (12), for example, found that mass media influences were more substantial than those provided by family members, peers, and schools. In his pilot study of high school students’ sources of information about a variety of drugs (including alcohol), Lamarine (13) discovered that the majority of students cited school, magazines, books, television, movies, and radio as their *initial* sources of information about alcohol. Much less frequently cited by respondents in this study were friends, parents, siblings, or other relatives. Similar findings were obtained by Casswell et al. (14) in their study of 8- and 9-year-olds and by Mizrahee et al. (15) in their study about drug information sources among adolescents. Austin and Nach-Ferguson (16) found that children aged 7–12 were equally likely to cite television and their parents as sources of their information about alcohol.

Moreover, several media effects researchers have also reported associations between television viewing behaviors and alcohol use behaviors among young people. For example, Tucker (17) reported that adolescent boys who were classified as heavy viewers of television drank more alcohol than adolescent boys who watched less television. Kulick and Rosenberg (18) showed that drinking-positive messages in films led older teenagers to develop greater expectancies about alcoholic beverages and greater intentions to drink. Kotch et al. (19) found that male children – but not female children – who had been exposed to a videotape containing drinking scenes in a television program subsequently reported more good things about alcohol than their peers who had been exposed to the same videotape but with the drinking scenes deleted from it. Neuendorf (20) reported that, among younger adolescents, greater exposure to television was associated with more favorable attitudes toward drinking. Based on their analysis of alcoholic beverage content in popular movies, Sargent and colleagues (21) concluded that characters’ use of alcohol in films was an independent risk factor for early-onset alcohol consumption in younger adolescents. Reviewing the literature on the effects of the media on subsequent substance use/abuse, Nunez-Smith and colleagues (22) concluded that 83% of media effects studies showed a link between media exposure and alcohol use. A similar conclusion was reached by Anderson and colleagues (23) in their review of published studies of the effects of alcoholic beverage advertising on adolescent alcohol use.

Given that (1) people start experimenting with alcohol while they are young, (2) beliefs and attitudes about alcoholic beverages and drinking precede young people’s first experiences using alcohol, (3) studies have shown that the mass media are among the earliest and most important sources of young people’s information about alcohol, (4) people are affected by the media messages to which they are exposed^1^, (5) young people appear to be more susceptible to the media messages to which they are exposed than their older counterparts are^2^, *and* (6) visual media like television, film, and video have been shown to be more influential than non-visual media forms^3^, it is important to learn more about the specific kinds of alcohol-related messages conveyed by the media to which young people are exposed if we wish to understand how they develop the notions they have about alcoholic beverages and alcohol use. Thus, the present study focuses on providing information about the messages conveyed about alcoholic beverages and drinking in a medium to which young people are exposed fairly heavily from an early age (i.e., animated cartoons).

It is worth pointing out that scholars have not paid much attention to the kinds of messages that the media convey about alcohol, with virtually no attention having been given to the alcohol-related content of programing aimed at young people. Here is a brief summary of what the alcohol-in-the-media literature *has* reported:

The consumption of alcoholic beverages on television is widespread, leading one author to comment that “alcohol is… inescapable on television” (24, p. 113) and that “television is the largest single source of information about drinking” (25, p. 21). Anywhere from 55 to 81% of all prime-time programs show or mention the use of alcohol (26–29) and 35–40% of all prime-time characters actually drink on-screen (30, 31). Tickle et al. (32) noted that anywhere from one-sixth to one-half of all movies contain at least 2.5 min of alcohol consumption, depending upon the movie’s rating as PG (17%), PG-13 (26%), or R (50%). Dal Cin et al. (33) reported that 83% of popular movies included alcohol consumption, and that the average American teenager is exposed to more than five full hours of alcohol consumption in the films that he/she sees. Forty percent of teenaged characters in top-grossing films were shown to consume alcohol and these drinkers typically were not shown to suffer any negative effects or adverse consequences from their drinking (34). Forty percent of all alcohol use on TV involves heavy drinking, and an additional 18% of all drinking acts involve chronic drinkers (35). Despite this, only 1–2% of all drinkers on television are supposed to be alcoholics or problem drinkers (31, 36) and the harmful effects that can result from drinking are mentioned only rarely (28, 31). In most (61%) episodes of televised drinking, the two main reasons given for the consumption of alcoholic beverages are to help characters deal with a crisis situation or to ease tension (35, 37). Alcohol use for hospitality, celebration, and enjoyment occurs with some frequency as well (27, 36). On television, rarely is it considered acceptable to refuse drinks; and disapproval of drinking, when expressed, is usually mild (35).

Thus, the message that is conveyed about alcoholic beverages is that it is okay, if not expected, for people to drink (34, 38). Heavy drinking also seems to be perfectly acceptable on TV, especially since virtually none of the heavy drinkers is shown to have significant drinking problems (29). There is, overall, a mixed message provided with regard to alcohol consumption (39). The mentality surrounding the use of beverage alcohol on television (i.e., that drinking and heavy drinking are acceptable, and that it is not truly considered permissible to refuse a drink) is aptly described by the term dysfunctional alcohol use (40) – a type of cultural attitude toward drinking that usually is associated with the subsequent development of alcohol problems. Recent experimental evidence among adults has surfaced, showing that exposure to alcohol-related content in television programing and in television commercials leads to increased alcohol consumption (41).

It is important to note, however, that the preceding studies are based on programing intended for *adult* audiences. Thus, despite the findings of this body of research, we still have very limited data on the portrayal of alcoholic beverages in programing aimed at *child* audiences. This is the subject of the present study. Here, we attempt to provide answers to the following research questions: (1) What is the prevalence of alcohol-related content in animated cartoons produced since 1930? (2) How, if at all, has that prevalence changed over the course of time? (3) What kinds of things do animated cartoons tell viewers about alcoholic beverages, alcohol use, and alcohol abuse?

## Materials and Methods

### Sampling strategy

This study is based on an examination of the content of animated cartoons. For the present study, only animated *cel* cartoons are included in the sample (e.g., Bugs Bunny, Popeye, Mighty Mouse, Yogi Bear). This eliminates from the present study such types of animation as claymation (e.g., Gumby and Pokey, the California Raisins), pixilation (the type of animation usually seen at the end of *The Benny Hill Show*), and puppet animation (e.g., *Davey and Goliath*, George Pal’s *Puppetoons*). The cartoons chosen for the study sample were selected randomly from among *all* cartoons produced between the years 1930 and 1996 by *all* of the major animation studios. Before drawing the final sample of cartoons that would be viewed and coded for this work, the researchers had to develop a comprehensive and inclusive sample frame of cartoons produced by the aforementioned animation studios. Published filmographies (42, 43) provided the authors with a great deal of this information. When a particular studio’s animated cartoon productions were not listed in the preceding sources or when the published listings for a specific company’s animated cartoon productions were not entirely up-to-date, the animation studios themselves were contacted and asked to provide comprehensive episode-by-episode lists of animated cartoons they had produced. This was done, for example, with studios like Hanna-Barbera, Marvel, Sunbow, Filmation, and a few others.

The origination date for this research (1930) was chosen for four reasons: (1) many major animation studios had begun operations by that time, (2) the era of silent cartoons had virtually ended, (3) cartoons produced prior to 1930 are not very accessible today, and (4) many cartoons produced during the 1930s are still broadcast on television and/or available for viewing on DVD or home video. Due to the fiscal constraints of the funding program, only animated cartoons with a total running time of 20 min or less were included in the sample frame.

A stratified (by decade of production) random sampling procedure was used to ensure that cartoons from all decades were represented equally in the study sample. This stratification procedure was necessary because very different numbers of cartoons have been produced during different decades (e.g., many more were produced during the 1980s than during the 1930s), thereby leading to the risk that a general random sample (as differentiated from this study’s stratified random sample) might have led to an overrepresentation of certain decades during which greater- or lesser-than-average numbers of alcohol-related portrayals were provided.

Cartoons on the sample list were obtained via numerous methods, including purchases of commercially available videocassettes from retail outlets, rentals of commercially available videocassettes from video rental outlets, videocassette purchases from catalog distribution companies, 16 mm film purchases from private animation collectors, and trading with private animation collectors to obtain VHS-format copies of needed cartoons in exchange for providing them with copies of cartoons they desired for their personal viewing/collections. In many instances, copies of the sample-listed cartoons needed for this project were provided by the animation studios themselves, in an effort to cooperate with this research study. Some of the cartoons on the sample list were taped from network television and cable television stations, too, although this was done primarily when other sources for obtaining these cartoon episodes were cost prohibitive or not readily available to the researchers. Recognizing that some cartoons appearing on television – particularly older cartoons that were originally made for showing in movie theaters prior to motion picture main attractions – have been edited for content, every effort was made to guarantee that the copies of cartoons obtained via taping from the television were complete and unedited.

### Data collection

This study uses a content analysis approach to examine the types of messages that cartoons provide about alcoholic beverages and drinking. Data collection for this research entailed viewing the cartoons contained on the project’s sample list and recording detailed information on predesigned, pretested, pilot tested, fixed-format coding sheets. Prior to beginning their viewing and coding work for this study, research assistants underwent an intensive training that familiarized them with the data that the study strived to collect, the rationale underlying the coding of each piece of information, and the decision-making procedures that should be used when recording information from each cartoon. To make sure that all people involved in the viewing/coding (i.e., data collection) process implemented the decision-making procedures in a similar manner, intercoder reliability coefficients were calculated periodically throughout the project. Reliability estimates consistently have fallen above 0.80 for all major measures, and were at least 0.90 for all of the variables used in the analyses reported in the present article, indicating a very high level of intercoder reliability for this research.

To understand the information that this study contains, it is best to conceptualize the database as consisting of three smaller datasets. Dataset No. 1 focuses on the cartoon itself as the unit of analysis and contains macro-level variables that provide prevalence-type information. Among several others, this dataset includes such measures as the cartoon’s length; number of characters of each gender, race, age group, and so forth; number of major and minor characters using alcohol; number of alcohol-related references made in the cartoon; and number of depictions of alcoholic beverages in the cartoon. This dataset facilitates analyses indicating the rate at which alcohol use portrayals are provided in the cartoons (i.e., there are *X* number of drinking-related messages for every *Y* min of viewing time), the proportion of all cartoons containing different types of alcohol use-related content, and so forth. *N* = 1,221 for this dataset.

Dataset No. 2 focuses on the major characters in each cartoon [regardless of whether they are human characters, animals, personified inanimate objects (e.g., cars with the ability to growl or dance, telephone poles given human-like abilities to see or hear or sing), monsters, ghosts, etc.], providing detailed information that is of value when trying to interpret the types of messages that cartoons provide about who it is that uses alcohol. This dataset contains information about each major character’s gender, age, race, ethnicity, marital status, level of intelligence, attractiveness, physique, occupational status, level of goodness or badness, and other demographic-type and descriptive information. In addition, Dataset No. 2 consists of data about the number of acts of violence, aggression, and prosocial behaviors (and limited information about the types of these behaviors involved) that the characters have committed. This dataset’s information is useful for examining such things as whether substance users are more likely to be male or female, old or young, attractive or unattractive, intelligent or unintelligent, violent or non-violent, prosocially oriented or not prosocially oriented, “good guys” or “bad guys,” and so forth. *N* = 4,201 for this dataset.

Dataset No. 3 provides detailed information about each portrayal of alcohol use. Such information as the type of alcohol used, the amount used, the effects – if any – that were shown to result from drinking, descriptive information about other characters shown to be present when the alcohol use occurred, the purported reason(s) why the drinking was taking place, and the location and social context of the drinking use were all captured in this dataset. *N* = 678 for this dataset.

### Operational definitions of some key concepts

In this research, codes were reserved for four types of alcoholic beverages. The first type of alcohol was beer, which included beer, ale, and stout. The second type is coded was wine, which included wine, champagne, port, and sherry. The third category was distilled spirits, which included liqueurs, all types of mixed drinks, and shots of “hard liquor.” Finally, generic alcohol was coded, which entailed those situations in which a beverage was portrayed to be alcohol even though its specific type was indeterminable based on the cartoon’s content. Usually, generic alcohol was identifiable as an alcoholic beverage by virtue of the alcoholic beverage container being marked with an “XXX,” the shape of the beverage container, or based on the effects shown to result from the use of the liquid (e.g., hiccupping).

This study entailed recording three types of alcohol-related content: alcohol use; depictions of alcoholic beverages; and references to alcoholic beverages, drinking, and/or the effects of consuming alcohol. To be coded as an act of alcohol *use*, the character must have an alcoholic beverage in his/her/its mouth or be trying to ingest alcohol. No particular amount had to be taken in order for use to be coded, as long as the intent/attempt-to-use criterion just mentioned was met. *Depictions* of alcoholic beverages are defined as instances in which the cartoon showed the actual beverage in question without also showing it being ingested. For example, if a cartoon were to show a bottle of beer lying on a table but no character drank from it, this would be coded as a depiction of alcohol. Finally, *references* to alcoholic beverages, drinking, or the effects of consuming alcohol are defined as those situations in which a cartoon or a cartoon character mentions an alcoholic beverage type, using an alcoholic beverage, or the effects resulting from drinking when alcohol itself neither was used nor shown in the cartoon. For example, if a character were shown to have a hangover and comment to another character that he couldn’t believe that he drank so much the night before, this would be considered a reference to the effects of drinking, since the alcoholic beverage itself is not shown and its use is not portrayed.

One of the key focal points of this research is examining what “types” of characters, based on their gender, race, age, “goodness” or “badness,” among other characteristics, are shown to be alcohol users. The operational definitions of these concepts are, in most instances, quite complex to describe succinctly, since numerous coding rules were developed for each construct to guarantee high levels of intercoder reliability. Rather than provide detailed explanations of each construct’s operational definition here, readers who wish to learn more about the specific ways in which these concepts were defined are encouraged to contact the lead author for additional information.

### Statistical analysis

All statistical analysis undertaken in conjunction with this research was performing using the Statistical Analysis System (SAS) software, version 9.1.3. Prior to undertaking any analysis of the project data, the appropriate power analyses/computations were conducted. All statistical analysis was performed with a minimum power of 0.8, which is the standard that is generally accepted for use in the social sciences (44). Many of the statistics that are reported in this article are purely descriptive. Analyses pertaining to changes that have occurred over time in cartoons’ content or to the prevalence of certain types of content entailed the use of simple regression. This was deemed appropriate since both the independent variable (i.e., year of production) and the dependent measures in question (e.g., proportion of all cartoons containing alcohol-related content, mean number of characters shown to be drinking or speaking about alcohol) were continuous. It should be noted that the changes-over-time data were also tested for curvilinearity by using multiple regression analysis, adding squared and cubed measures of the independent variable (year of production) to the “ordinary” production-year variable. Chi-square tests were used to determine whether characters that used alcohol differed from those who did not based on various demographic-type variables. Chi-square tests were selected because the demographic measures in question (e.g., gender, age classification, racial group membership) were categorical and the alcohol user measure was dichotomous. Throughout this article, results are reported as being statistically significant whenever *p* < 0.05.

## Results

### Overall prevalence information

Table 1 provides a summary of prevalence information for the various types of cartoons’ alcohol-related content, averaged over time. *Alcohol use* was portrayed in 3.2% of the cartoons coded. The average cartoon with any characters using alcohol contained approximately two (mean = 1.9, SD = 2.1) such characters consuming alcohol. Of the different types of alcoholic beverages that could be consumed, beer was the most common, accounting for 35.6% of all alcohol use in the cartoons studied. Next most frequently consumed were, respectively, wine (27.8%), distilled spirits (25.6%), and generic alcoholic beverages (11.1%).

**Table 1:** **Prevalence of alcohol-related content in cartoons, averaged over time**.

Type of alcohol-related content	% of cartoons	Number per cartoon with content	Number per viewing hour	Number per viewing week
Depiction	5.6	2.9	1.1	12.3
Reference	3.4	1.9	0.5	5.0
Actual consumption	3.2	1.9	0.4	4.7
Any alcohol-related content	9.3	3.1	2.0	22.0

*Depictions of alcoholic beverages* were found in 5.6% of the cartoons coded. When a cartoon depicted an alcoholic beverage without also showing it being consumed, the average cartoon did so nearly three times (mean = 2.9, SD = 5.4). *Alcohol-related references* involving neither consumption nor depiction of the beverage were found in 3.4% of the cartoons coded, with the average cartoon with any alcohol-related reference approximately two references to it (mean = 1.9, SD = 2.1). Overall, about 1 cartoon in 11 (9.3%) incorporated alcohol into its plot in one way or another. When alcohol was brought into a cartoon’s storyline, it showed up an average of three times (mean = 3.1, SD = 5.6) during the course of the cartoon.

Another way to examine the preceding prevalence information – one that helps to put these prevalence figures into their proper perspective – is to convert it into rates presenting the number of alcohol-related messages per hour of viewing time. When computing an hour of viewing time, we assume that a typical hour of television viewing of children’s programing consists of 45 min of programing and 15 min of commercial time. It is, therefore, a conservative estimate of the actual prevalence of alcohol-related content in the cartoons since, by recently established federal law, programs broadcast during the hours considered “children’s programing time” must contain no more than 15 min of commercials. Our data indicate that the typical hour of cartoon viewing will expose viewers to two alcohol-related messages (mean = 2.0, SD = 12.8). Over the course of a “typical” week’s television and video viewing [approximately 18.3 h spent by children (45)], and given the proportion of this viewing time that is spent watching television during children’s programing hours [60–80% (46)], a reasonable, mid-range estimate is that the “average” young person is exposed to approximately 22 alcohol-related messages each week just from the animated cartoons that he/she watches.

### Changes in prevalence

**Figure 1:**
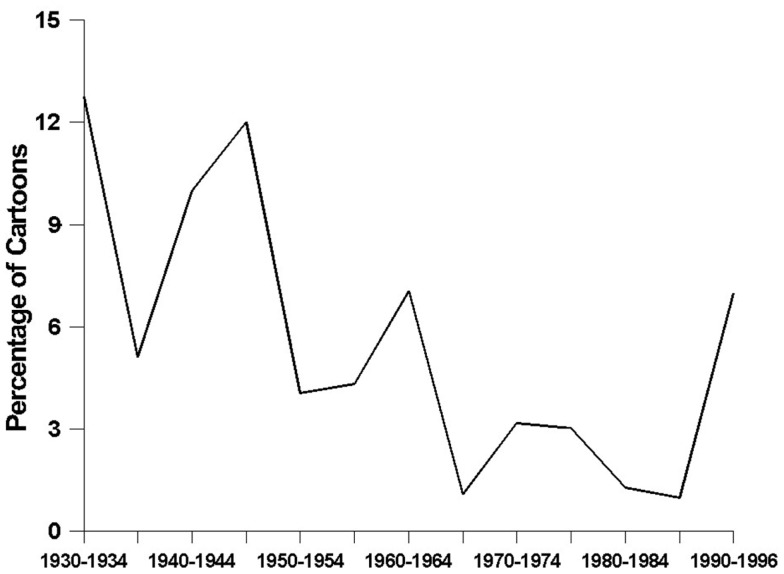
**The prevalence of alcohol-related depictions**.

**Figure 2:**
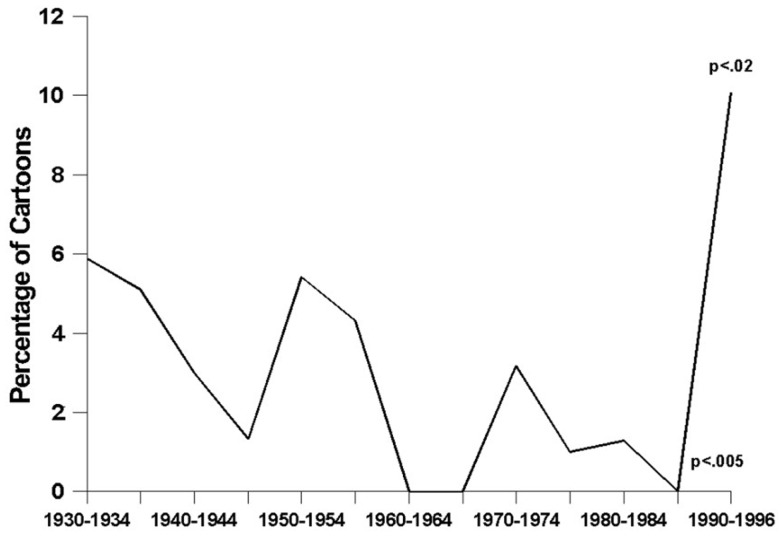
**The prevalence of alcohol-related references**.

**Figure 3:**
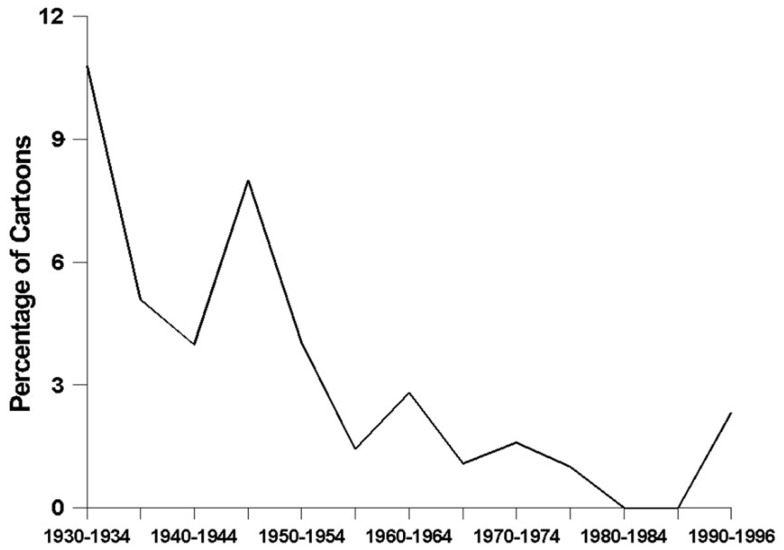
**The prevalence of alcohol use among major and minor characters**.

*Depictions of alcoholic beverages* have become significantly less frequent over time (*F*_2, 1218df_ = 5.29, *p* < 0.006) (see Figure 1). The data revealed a nearly-significant curvilinear relationship, with steady declines in the prevalence of alcohol-related depictions from the 1930s through the 1980s (*t* = 2.12, *p* < 0.04), followed by a noticeable increase during the 1990s (*t* = 1.77, *p* < 0.08). Figure 2 shows how the prevalence of *alcohol-related references* has changed over time – a relationship that was found to be statistically significant and curvilinear (*F*_2, 1218df_ = 6.16, *p* < 0.003). Essentially, alcohol-related references declined in frequency fairly steadily from the 1930s until the 1980s (*t* = 2.87, *p* < 0.005), and then increased very sharply to their highest levels ever during the 1990s (*t* = 2.57, *p* < 0.02). As time has gone on, cartoons have been containing significantly fewer *characters using alcohol* (*F*_1, 1219df_ = 14.77, *p* < 0.0001), a decline that is demonstrated quite clearly in Figure 3. This finding was obtained for major (*F*_1, 1219df_ = 10.93, *p* < 0.001) and minor (*F*_1, 1219df_ = 8.21, *p* < 0.005) characters alike.

**Figure 4:**
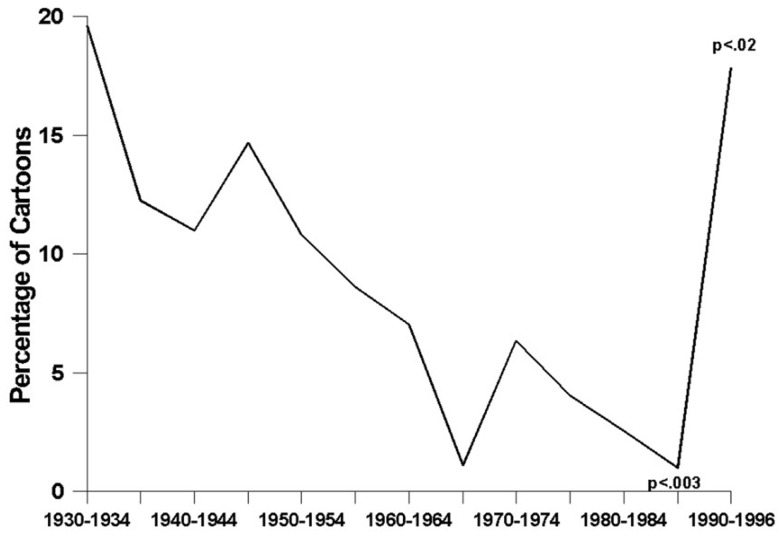
**Any alcohol-related content in animated cartoons**.

Given the preceding, it is not surprising that the overall presence of alcohol (including alcohol use, depictions of alcoholic beverages, and alcohol-related references) has declined significantly over the years (*F*_2, 1218df_ = 9.75, *p* < 0.0001). This is shown in Figure 4. These analyses revealed that, during the 1990s, after a fairly steady, 60-year-long decline in cartoons’ alcohol-related content (*t* = 2.98, *p* < 0.003), there was a sharp, statistically significant increase (*t* = 2.51, *p* < 0.02) that brought the prevalence of any alcohol-related content to nearly its highest level ever.

### Information conveyed about drinking

In light of the frequency with which young people are being exposed to cartoons’ alcohol-related messages, it becomes important to study what exactly they are being told about alcoholic beverages and drinking. Toward this end, we now turn our attention to the effects that are shown to result from alcohol use. From there, we provide information about the drinking context and about what “types” of characters are shown to be present whenever alcohol use takes place. We conclude this section by presenting information about which characteristics are associated with drinking and which are not.

#### Effects of drinking

In nearly one-half (46.7%) of all instances in which alcohol use occurs, drinking is shown to result in no physical effects of any kind. When effects *are* shown to result from ingesting alcohol, only two are portrayed in at least one-half of all instances: hiccupping (shown as a consequence of drinking in 75.0% of these portrayals) and loss of coordination (which occurred in 70.8% of these instances). All other effects resulting from drinking were shown quite infrequently. Examples include becoming unconscious (16.7%), increased happiness or sociability (12.5%), increased relaxation (4.2%), becoming interested in another character sexually (6.3%), arguing or physically fighting with other characters (4.2%), increased sleepiness (4.2%), coughing (2.1%), hallucinating (2.1%), increased rowdiness (2.1%), becoming sick (0%), among others. In all, characters were equally (un)likely to experience positive effects as a result of their alcohol use (e.g., increased happiness, greater relaxation, etc.) as they were to experience negative effects from their alcohol use (e.g., falling unconscious, physically fighting with others, etc.), as both categories of effects were observed in 20.8% of all instances involving alcohol consumption.

#### Context of alcohol use

Table 2 provides information about selected characteristics of the sample population of cartoon characters in general and how those characteristics compare to the traits of characters that were present when some character was consuming alcohol. Nearly one-half (46.7%) of all instances of alcohol use in the cartoons occurred while the drinker was alone. When other characters were present during the drinking occasion, they were 2.4 times more likely to be male than female. This gender disparity is less than one-half than that seen among cartoon characters as a whole, which is approximately 5.1:1.

**Table 2:** A comparison of selected characteristics of the cartoon sample overall and the characters that were present when alcohol was consumed in cartoons*.

Characteristic	% of entire research sample	% of characters present during drinking
Gender
Male	84.1	93.3
Female	15.9	6.7
Race
Caucasian	91.6	83.1
Non-Caucasian	8.4	16.9
Age group
Infant, child, adolescent	19.8	2.0
Adult	76.6	93.4
Elderly	3.6	4.6
Physical attractiveness**
Unattractive/ugly	5.9	5.4
Average looking	90.1	93.3
Attractive/good looking	4.0	1.3
Intelligence**
Below average/dumb	3.8	0.0
Average	92.4	99.6
Above average/smart	3.8	0.4

*Characters coded as “undeterminable” for any particular category have been omitted from these computations.

**When reviewing the statistics for physical attractiveness and intelligence, readers are urged to keep in mind the coding rules for defaulting to “average” and the requirements imposed for categorizing characters as something other than “average.”

With regard to race, all of the characters with a codable race^4^ that were present during situations in which alcohol was used were Caucasian. This is particularly interesting in light of the fact that 8.5% of the characters appearing in the cartoons were members of racial minority groups.

Regarding age, 2.1% of the characters present when alcohol was consumed were infants, children, or adolescents. This is much lower than the 21.4% figure representing the proportion of all characters in the cartoons that fall into these age groups. Another 1.4% of the cartoon characters that were present when alcohol was used were classified as elderly, which is somewhat smaller than the proportion of cartoon characters that were coded as elderly (3.4%).

In terms of the physical attractiveness of the characters that were portrayed in the presence of alcohol use, 11.8% of them were coded as above average in attractiveness – nearly triple the percentage of all cartoon characters that were coded as good looking (4.4%). Conversely, only 4.2% of those present when one character consumed alcohol were classified as being unattractive, which is comparable to the 5.4% of characters that coded were classified as being physically unattractive.

The locations and social contexts of drinking episodes were highly varied in the cartoons coded. Most commonly, alcohol use occurred in outdoor settings (52.2%), with nearly one-half of *these* settings (44.7%) entailing rather generic outdoor locations in the country (e.g., by the roadside, in a meadow, in a forest). When drinking was portrayed as an indoor activity, it occurred most often in characters’ homes (accounting for 46.5% of all indoor drinking locales, or 22.2% of all locales where alcohol use occurred) or in bars, taverns, or saloons (accounting for 30.2% of all indoor drinking locales, or 14.4% of all locales where alcohol was consumed). Somewhat surprisingly, there were no portrayals of alcohol use in conjunction with sporting events or such occasions as birthday parties, weddings, anniversary celebrations, or other holiday celebrations.

#### Reasons for drinking

The purported reasons for cartoon characters’ use of alcohol were rather varied. The single most common explanation of cartoon characters’ use of alcohol was that they simply enjoyed the taste of alcohol or because they liked to drink, which accounted for 12.2% of all use portrayals. The next most common reason for using alcohol was to become drunk (7.8%), followed by using alcohol to be more sociable or “to be part of the crowd” (5.6%). It is worth mentioning that in 40.0% of all alcohol use portrayals, drinking occurred for no reason whatsoever. That is, based on the cartoon’s events and the context in which the alcohol use occurred, there was no inference to be made as to why the drinking was happening.

#### Characteristics associated with alcohol use

Table 3 presents summary information on various traits associated with characters’ use versus non-use of alcohol. In terms of which “types” of characters used alcohol and which did not, several significant differences were found between drinkers and non-drinkers. Characters that used alcohol engaged in nearly twice as many antisocial behaviors (including physical aggression, verbal aggression, lying, and violence) as their non-drinking counterparts did (4.9 acts versus 2.6 acts; *t* = 3.84, *p* < 0.0001). In great part, this finding was “driven” by the substantially greater perpetration of violence among drinking characters than among non-drinking characters (4.1 violent acts versus 1.5 violent acts; *t* = 5.13, *p* < 0.0001), although alcohol users were somewhat less verbally aggressive than their non-drinking counterparts (0.1 acts of verbal aggression versus 0.5, *t* = 2.07, *p* < 0.04). Caucasian characters were far less likely to be alcohol users than non-whites were (OR = 0.15, CI_95_ = 0.04−0.52, *p* < 0.0006). Elderly characters were much more likely than their child, adolescent, or adult counterparts to ingest alcohol (OR = 8.54, CI_95_ = 3.59−20.30, *p* < 0.0001). Alcohol users were more than three times as likely as non-users to be portrayed as physically unattractive compared to characters that did not use alcohol (OR = 3.67, CI_95_ = 1.75−7.66, *p* < 0.0002). In terms of their overall classification as a “good guy” versus a “bad guy” (versus those of mixed traits and those classified as being neither good nor bad), alcohol users were twice as likely as non-users to be classified as “bad guys” (OR = 2.15, CI_95_ = 1.21−3.82, *p* < 0.008).

**Table 3:** Demographic/descriptive and behavioral characteristics associated with being a non-user versus a user of alcohol (major characters only)*.

Characteristic	Non-users of alcohol	Users of alcohol
Gender (%)
Male	15.9	14.8
Race (%)
Caucasian	91.2	60.0
Age (%)
Elderly	3.4	23.3
Intelligence (%)
Below average/dumb	3.9	0.0
Above average/smart	3.8	4.3
Physical attractiveness (%)
Below average/ugly	5.8	19.2
Above average/attractive	4.0	0.0
“Good guy”/“bad guy” (%)
Bad guy	34.6	53.2
Good guy	25.6	6.4
Prosocial behaviors (mean number of acts)
Provide physical assistance	0.23	0.34
Provide financial assistance	0.01	0.13
Provide knowledge or information	0.06	0.00
Show concern	0.28	0.13
Compliment	0.21	0.04
Total – all prosocial behaviors	0.79	0.64
Antisocial behaviors (mean number of acts)
Verbal aggression	0.47	0.11
Physical aggression	0.49	0.57
Lying or deception	0.12	0.11
Violence	1.50	4.11
Total – all antisocial behaviors	2.57	4.89

*Characters coded as “undeterminable” for any particular category have been omitted from these computations.

Drinkers and non-drinkers did not differ, from a statistical point of view, in terms of their gender breakdown ($\chi_{\text{1df}}^{2}\;=\;$0.02, n.s.), intelligence ($\chi_{\text{2df}}^{2}\;=\;$1.90, n.s.), or the number of prosocial behaviors committed (*t* = 0.68, n.s.).

## Discussion

Before discussing the implications of our main findings, we would like to acknowledge a few potential limitations of the present study. First, this research was based on animated cartoons with running times of 20 minutes or less, thereby excluding longer-form animated cartoons from consideration. We do not know whether or not short-form and long-form animated cartoons are similar to one another with respect to the types of messages they convey, and therefore cannot assess the extent to which the exclusion of the latter may affect this study’s findings. Conducting research such as ours with the longer cartoons would be a worthwhile endeavor for future researchers to undertake. Second, our sample ends during the middle-1990s. It would be helpful and, we believe, interesting to have this research extended to the present, so that the most up-to-date trends possible are studied and analyzed. Third, as with any content analysis research study, some scholars might prefer to see different operational definitions of the key concepts used. There is no “gold standard” in content analysis research with regard to defining major versus minor characters, alcohol-related references or depictions, and so forth. The definitions that we adopted were chosen on the basis of common sense, so that they would foster face validity, and on the basis of simplicity and clarity of implementation, so that they would maximize interrater reliability. We believe that our operational definitions are well-conceptualized and justified; but as with any content analysis study, there is no way to know the extent to which the use of different definitions might have led to different research findings.

Despite these potential limitations, we still believe that the present research has much to contribute to our understanding of cartoons’ messages about alcoholic beverages and drinking. First, the cartoons that young people (and adults too!) watch contain a modest amount of alcohol-related content. Although the frequency with which alcohol-related messages are portrayed has declined over the years (give or take the substantial upsurge in alcohol-related content that we observed in cartoons produced during the 1990s), readers should bear in mind that many of the cartoons produced during the 1930s, 1940s, and 1950s (when alcohol-related content was at its peak) are still broadcast on network and cable television today *and*, increasingly, are being released on home video and DVD for private use, as well as being broadcast on cable stations such as Boomerang. Thus, although alcohol-related messages were provided less often in cartoons produced during the 1970s and 1980s, such messages remain widely available in the cartoons that people watch. Our finding that alcohol-related content is quite prevalent in animated cartoons is similar to the findings of several researchers (e.g., 24, 27, 29, 32, 33), whose research, based on programing aimed at adult audiences, also revealed that drinking-related messages are ubiquitous in the electronic media. Our discovery of a fairly high prevalence of alcohol-related content in cartoons even more closely resembles that obtained by Penkoff (47) in her content analysis of the alcohol-related content in Walt Disney cartoons.

Of even greater interest and importance, we feel, is the information that this study can provide about the things that young people learn about alcoholic beverages and drinking when they watch animated cartoons. Overall, they are provided with mixed messages about alcohol use. On the negative side, cartoon characters that use alcohol are more violent, more likely to be shown to be physically unattractive, and more likely to be depicted, overall, as “bad guys” compared to cartoon characters that did not use alcohol. Conversely, cartoons convey quite clearly that using alcohol is associated with a variety of socially desirable qualities, and they present this message in many ways: Males, who our culture values more and stigmatizes less than females, are more than three times more likely than females to be shown to be present when alcohol is used. Similarly, Caucasians, who our culture favors over people of color, also have a much-greater-than-average chance of being shown to be present when alcohol is used. Likewise, physically attractive characters are three times more likely to be associated with alcohol-drinking characters than physically unattractive characters are – once again remaining consistent with our cultural emphasis on beauty. All of these messages (and others similar to them) convey and reinforce an important notion to viewers: “good,” “desirable,” “culturally valued” people are the ones who are associated with alcohol users. This is the type of socialization message that is likely to make young people want to experiment with alcohol so that they, too, can be part of a socially desirable group of people. It is the same kind of message that Johnston et al. (48, pp. 3–4) say pertains to media portrayals of tobacco use:

Cigarette smoking is continually associated with social success, sexual attractiveness, a healthy demeanor, exciting sporting activities… and so on. What else could an American adolescent want?

Reinforcing these notions about the positive aspects of using alcohol are the settings in which cartoons show alcohol consumption taking place. Typically, alcohol is used in relaxation-oriented contests and outdoor surroundings that are peaceful, pleasant, and enjoyable, such as meadows, forests, or in conjunction with picnics. While there is no inherent danger in such messages, they *do* serve to reinforce the ideas that drinking is good and that it can lead to feeling soothed and relaxed. This type of message exemplifies Bales’ (49) concept of hedonistic alcohol use, which some researchers (50, 51) have found to be linked to increased consumption.

Regarding why cartoon characters purportedly use alcohol, utilitarian rationales (49) seem to underlie a majority of the instances in which alcoholic beverages are consumed. Drinking can be classified as utilitarian when alcohol is consumed so that the drinker derives some direct, personal benefit from the alcohol use. In the cartoons, utilitarian-type drinking was manifested by characters that drank specifically to: become drunk, become more sociable, make sure that they were not left out of the crowd’s activities, relieve thirst, and/or become more energetic. Combined, these situations accounted for about one-half of all instances in which any reason was implied as to why the character in question used alcohol.

One final thing that we would like to highlight from our findings – something that is not very evident from the data presented in this article – is that cartoons with alcohol-related content that does not glamorize drinking or make alcohol use seem appealing tend to present messages about alcoholic beverages and drinking that normalize these things. This is perhaps most evident when one looks at the frequency (which is such that it accounts for approximately one-half of all alcohol-related content in animated cartoons) and the nature of cartoons’ alcohol-related depictions and references. Regarding the latter, we have found that, in most instances in which cartoon characters mention alcohol or the effects of drinking without actually consuming alcohol, and in most instances in which alcoholic beverages are shown on-screen without being used, the inclusion of these references and depictions is unnecessary – some might even call it gratuitous – to the flow and development of the cartoon’s storyline. Two examples from cartoons that from later in the study’s period of investigation will illustrate the point here:

**Example No. 1:** The 1993 Garfield cartoon entitled *DJ Jon* deals with one character becoming a radio disk jockey and how his infatuation with the glamor of the job causes him to ignore his pets. Until the last 15 s of the cartoon, there is no involvement of alcoholic beverages, drinking, or references to alcohol. In the final fade-to-black of the cartoon, however, the characters exit the radio station and walk down the street. Along the way, they pass several buildings, only one of which is labeled in any way… with the word “BAR” appearing in flashing neon. The characters walk past the bar, do not appear to notice it or comment on it, and the cartoon ends.

**Example No. 2:** The 1993 Animaniacs cartoon entitled *Can’t Buy a Thrill* shows husband and wife hippopotamuses seeking interesting and exciting things to do to perk up their marriage. They begin by bungee jumping (which they find mundane), then proceed to diving with sharks (which they find boring), and then decide to participate in the annual running of the bulls in Pamplona, Spain (which they find exhilarating). Throughout all of their adventures in the cartoon, alcohol remains uninvolved… again until the last 15 s of the cartoon. At that time, both of the characters are shown toasting one another with umbrella-adorned presumed-to-be-alcoholic beverages, to congratulate one another on finding a suitable and thrilling adventure. After raising their glasses in celebration, the cartoon ends. No alcohol is consumed.

Both of these examples illustrate the preceding point quite well: The inclusion of the alcohol-related content in these particular cartoons was unnecessary, not really enhancing or facilitating the plots of these cartoons at all. Nevertheless, in both instances – instances that we feel represent a fairly common trend among the types of alcohol depictions and references seen in the animated cartoons we have been studying – the alcohol-related content was innocuous enough. It is cartoons’ frequent presentation of these unnecessary yet primarily benign messages about alcohol that leads us to conclude that, when they are not making alcohol seem appealing to viewers, cartoons make alcohol seem “normal,” “natural,” “harmless,” and “benign” to viewers. Perceptions such as these, while unlikely to induce young people to experiment with alcohol or to continue using it once their experimentation has begun, are, in our opinion, likely to foster the impression that there is nothing wrong with trying alcohol, nothing to be lost by drinking, and so forth. Perhaps this accounts, at least in part, for youths’ involvement with alcoholic beverages.

To the extent that one “buys into” the principal tenets of major theoretical paradigms such as social learning theory [see, for example (52, 53)], cultivation theory [see, for example (54–56)], and priming effects theory [see, for example (57, 58)], the aforementioned findings pertaining to alcohol use, alcohol depictions, and alcohol-related references are of especial significance. For example, social learning theory posits that people acquire their beliefs, attitudes, and propensity to engage in behaviors, directly based on first-hand experiences they have with others who exhibit particular behaviors *and/or* indirectly, based on what they observe others – including others appearing in the mass media – doing or saying. As Kunkel et al. (59, p. I-6) put it, “through the observation of mass media models the observer comes to learn which behaviors are ‘appropriate’ – that is, which behaviors will later be rewarded, and which will be punished.” Applied to the present study’s findings, social learning theory would predict that young people will learn a great deal about what “kinds” of people (e.g., good versus bad, attractive versus unattractive, intelligent versus unintelligent, etc.) use alcohol, when, and in what circumstances and contexts, and about the overall acceptability of drinking, just from watching what animated cartoon characters do and from being exposed to the repeated alcohol-related messages that these cartoons contain.

Similarly, cultivation theory states that media viewers’ perceptions of social reality will be shaped by extensive and cumulative exposure to media-provided messages. This theoretical model assumes that people develop beliefs, attitudes, and expectations about the real-world based on what they see and hear on television, on video, in film, etc. Subsequently, they use the beliefs, attitudes, and expectations they have developed to make decisions about how they will behave in real-world settings and situations. Again, Kunkel et al. (59, pp. I-11, I-13) put it well when they stated:

The media, in particular television, communicate facts, norms, and values about our social world. For many people television is the main source of information about critical aspects of their social environment… Whether television shapes or merely maintains beliefs about the world is not as important as its role in a dynamic process that leads to enduring and stable assumptions about the world.

In the context of the present study, then, cultivation theory would posit that animated cartoons serve as agents of socialization regarding what to think about alcoholic beverages and how to feel about using them. This would be particularly true for young viewers who are exposed rather heavily to cartoons’ alcohol-related messages. Given the generally positive messages that cartoons provide about alcoholic beverages and drinking, cultivation theory would predict that the cumulative effect of exposure to these messages would provide young people who lacked information about alcohol with “alcohol friendly” beliefs and attitudes toward drinking and, in the process, lead many of them to experiment with alcohol or to adopt mind sets that were conducive to the use of alcohol.

Although its relative newness makes it less well-known to researchers, priming effects theory is also quite relevant to this study and its findings. This theory posits that, when people are exposed to something in the media, ideas are generated and/or brought forth to the foreground of the person’s thoughts and memory. For a short period of time thereafter, these ideas remain active and easily accessible, and during that time, they bring other, related thoughts and memories to the foreground. Thus, the media-provided messages are priming the thought and memory processes. Priming effects theory also postulates that “viewers who identify with certain actors may be vividly imagining themselves as these characters and thinking of themselves as carrying out the depicted actions. Identification with characters in the mass media should activate high imagery thoughts and the subsequent priming of these thoughts might influence subsequent behavior” (59, p. I-8). In the context of the present study, priming effects theory would predict that alcohol-related messages to which viewers are exposed in the animated cartoons that they watch will be combined with other information they already have about alcoholic beverages, drinkers, drinking, etc. The former will draw out the latter, increasing its saliency to the person and the person’s actions. In this manner, priming effects theory posits, watching cartoons that contain alcohol-related content will lead many young people to think about alcohol, think about how well they personally can relate to the characters they are seeing on the screen, and consider whether or not they wish to use alcohol or become involved with alcohol in ways similar to those shown in the cartoons.

Ultimately, we believe that the frequent inclusion of alcohol-related content in animated cartoons, coupled with the frequently pro-drinking messages about alcohol use that the cartoons provide, combine to tell audiences that alcohol is a normal, positive aspect of life. Cartoons tell people that drinking only sometimes has an effect on the drinker *and* that many of the effects that are most likely to occur (e.g., hiccupping, increased happiness or sociability, increased relaxation) are positive in nature. This conclusion is quite similar to that reached by Penkoff (47). With these types of messages being most indicative of the kinds of things that people learn about alcohol from watching animated cartoons, it is not surprising that young people are interested in and willing to experiment with alcoholic beverages. With cartoons showing alcohol to be an acceptable, normal part of everyday living that is associated with traits that our culture values *and* by associating few truly negative consequences with alcohol use, why wouldn’t young people want to experiment with drinking?!

## Conflict of Interest Statement

The authors declare that the research was conducted in the absence of any commercial or financial relationships that could be construed as a potential conflict of interest.

## Footnotes

^1^Additional support for this statement can be found in (60–64), among many others.

^2^Interested readers may wish to consult (8, 65, 66) for more information about this notion.

^3^Additional information about this idea may be found in (24, 66–69).

^4^Many cartoon characters are racially indistinct and, as a result, are coded as “race is indeterminable” or “race does not apply to this character” in this study.
